# Order–disorder (OD) polytypism of K_3_FeTe_2_O_8_(OH)_2_(H_2_O)_1+*x*_

**DOI:** 10.1107/S2052520623009162

**Published:** 2023-11-07

**Authors:** Tobias Wolflehner, Berthold Stöger

**Affiliations:** a X-Ray Centre, TU Wien, Getreidemarkt 9, 1060 Vienna, Austria; Georgetown University, USA

**Keywords:** polytypism, OD theory, X-ray diffraction, tellurate

## Abstract

K_3_FeTe_2_O_8_(OH)_2_(H_2_O)_2_ was synthesized under hydrothermal conditions from Te(OH)_6_, FeSO_4_·7H_2_O and 85 wt% KOH in a 1:2:6 molar ratio. The crystal structure is built of a triperiodic network. One disordered water molecule per formula unit is located in a channel and can be partially removed by heating. Systematic one-dimensional diffuse scattering indicates a polytypic character, which is best described by application of the order–disorder theory. The major polytype is monoclinic with pseudo-orthorhombic metrics. It is interrupted by fragments of an orthorhombic polytype. The diffraction intensities are analyzed using structure factor calculations.

## Abstract 2

K_3_FeTe_2_O_8_(OH)_2_(H_2_O)_1+*x*_ crystallizes as an order–disorder structure where polytypes differ in the positions of the Fe and Te atoms. Diffuse scattering with broad peaks indicates correlated disorder.

## 1. Introduction

Single crystals of the hydrous potassium iron(III) tellurium(VI) oxide K_3_FeTe_2_O_8_(OH)_2_(H_2_O)_2_ were grown under hydrothermal conditions in an attempt to synthesize Fe analogues of K_2_Ni_2_TeO_6_, a fast potassium ion conductor with potential application as battery materials (Masese *et al.*, 2018 ▸). On heating, weakly-bound water is gradually lost, resulting in K_3_FeTe_2_O_8_(OH)_2_(H_2_O)_1+*x*_ (0 ≤ *x* < 1) with otherwise unchanged structure. The fully hydrated compound and the partially dehydrated products can be formulated as K_3_FeTe_2_O_8_(OH)_2_(H_2_O)_1+*x*_.

The title compounds feature order–disorder (OD) polytypism (Dornberger-Schiff & Grell-Niemann, 1961 ▸). This means that the structure is built of layers that can be stacked in different ways and all are locally equivalent. In particular, adjacent pairs of layers contact in such a way that the resulting pairs are geometrically equivalent. The OD theory thus interprets the common occurrence of polytypism and provides a unified description of families of OD structures.

Herein, a detailed OD description of K_3_FeTe_2_O_8_(OH)_2_(H_2_O)_1+*x*_ is given. The diffraction pattern is analyzed using structure factor calculations.

## 2. Experimental

### 2.1. Synthesis

A mixture of Te(OH)_6_, FeSO_4_·7H_2_O and 85 wt% KOH in a 1:2:6 molar ratio were introduced into PTFE inlays with ∼3 ml inner volume. The inlays were three-quarters filled with deionized water, introduced into steel autoclaves and placed in a pre-heated drying closet at 210°C. After three days, the autoclaves were cooled in air for ∼4 h. The solid residue was washed twice with water and twice with isopropanol and finally dried at 35°C in air to give single phase K_3_FeTe_2_O_8_(OH)_2_(H_2_O)_2_ according to powder X-ray diffraction. The product was obtained as a mixture of microcrystalline powder and tiny lath-shaped single crystals.

### 2.2. Single crystal diffraction

Single crystals of K_3_FeTe_2_O_8_(OH)_2_(H_2_O)_2_ were selected under a polarizing microscope. A 300 K dataset was collected on a Bruker KAPPA APEX II diffractometer system equipped with a Mo *K*α sealed tube, a CCD detector and an Oxford Cryosystems Cryostream 800. High temperature measurements and reciprocal space maps were collected on a STOE Stadivari diffractometer system equipped with a Mo *K*α microsource, a DECTRIS Eiger CdTe hybrid photon-counting (HPC) detector and a STOE Heatstream heating system. To reconstruct the diffuse scattering, a room temperature dataset with very long exposure times (120 s per degree) was collected, taking advantage of the practically zero-noise of HPC detectors.

Data were processed using the *APEX*4 suite (Bruker, 2022 ▸) and *X-Area* (STOE & Cie GmbH, 2021 ▸). Corrections for absorption effects were applied using the multi-scan approach followed by a spherical absorption correction implemented in *SADABS* (Bruker, 2022 ▸) and *LANA* (STOE & Cie, 2021 ▸). Data collection and refinement details are compiled in Table 1 ▸.

### 2.3. Structure solution and refinement

The unit-cell parameters strongly suggested an ortho­rhombic *I*-centered (*oI*) lattice. A first structural model was obtained using the dual-space methods implemented in *SHELXT* (Sheldrick, 2015*b* ▸) in the space group *Imma*. The structure was refined using *SHELXL* (Sheldrick, 2015*a* ▸). Non-hydrogen atoms were refined using anisotropic displacement parameters (ADPs).

The central atom of an [*M*O_6_] octahedron had to be modeled as an occupationally disordered Te/Fe site (labeled as Te1/Fe1) with an 1:1 occupation ratio. Refinements resulted in reasonable reliability factors {*R*[*F* ^2^ > 2σ(*F* ^2^)] ≈ 0.06}, however, the ADP tensors were highly anisotropic.

Since we suspected ordering of the Te/Fe site, we attempted structure solutions in the maximal subgroups of *Imma* with the same translation lattice, also called *translationengleiche* subgroups (Müller, 2013 ▸), namely *Im*2*a*, *I*2*ma*, *Imm*2, *I*2_1_2_1_2_1_, *I*2/*m*11, *I*12/*m*1 and *I*112/*a*. The lost symmetry operations survive in the crystalline edifice as twin operations and define the twin law that was applied in the subsequent refinements.

A satisfying structure model was only obtained in *I*2/*m*11. The Te/Fe site of the orthorhombic *Imma* structure split into two sites with distinctly different electron densities. Yet, both sites still had to be modeled as mixed Te/Fe sites (Te1:Fe1′ and Fe1:Te1′) with the overall Te:Fe ratio being fixed to 1:1. For convenience, the axes were permuted to the standard setting of monoclinic space groups (*b* unique). In this setting, the space group of the final model is *I*2/*m* and that of the orthorhombic parent structure is *Ibmm*.

H atoms were located from difference Fourier maps and the O—H distances were restrained to 0.870 Å. The H atoms of the disordered water molecule in channels of the structure could not be localized. The occupancy of the disordered water molecule refined to 1 within experimental precision for the 300 K dataset and to 43 (5)% for the dataset collected at 436 K.

A further dataset was collected at 489 K, though was of even worse quality and is therefore not presented here. Increasing the temperature to 540 K let to a decomposition of the crystal, as evidenced by planes of diffuse scattering normal to b* (though no formation of powder rings).

## 3. Results and discussion

### 3.1. Structure overview

**Figure 1:**
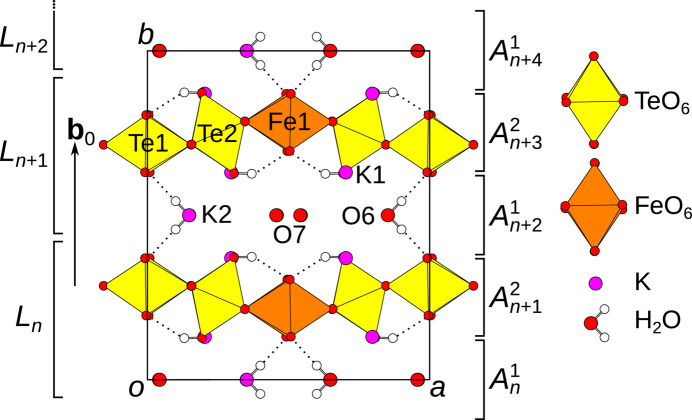
The crystal structure of K_3_FeTe_2_O_8_(OH)_2_(H_2_O)_2_ viewed along [001]. H (white), O (red) and K (pink) are represented by spheres of arbitrary radius, [TeO_6_] and [FeO_6_] units by yellow and orange polyhedra, respectively. Hydrogen bonds are indicated by dotted lines.

The crystal structures of K_3_FeTe_2_O_8_(OH)_2_(H_2_O)_1+*x*_ (Fig. 1 ▸) are built of wavy sheets of a FeTe_2_O_8_(OH)_2_ network extending in the (010) plane. The sheets are connected by K atoms and a water molecule (O6) located on a (010) reflection plane (see Table 2 ▸). Channels in the structure extending along [001] are filled with a varying number of water molecules (O7) depending on measurement temperature. Owing to the loss of water, the unit-cell volume decreases marginally from 300 K to 436 K (see Table 1 ▸). However, as expected, the overall density decreases on heating. The *a* parameter decreases by ∼0.3%, owing to a shrinking of the channels.

### 3.2. OD interpretation

**Figure 2:**
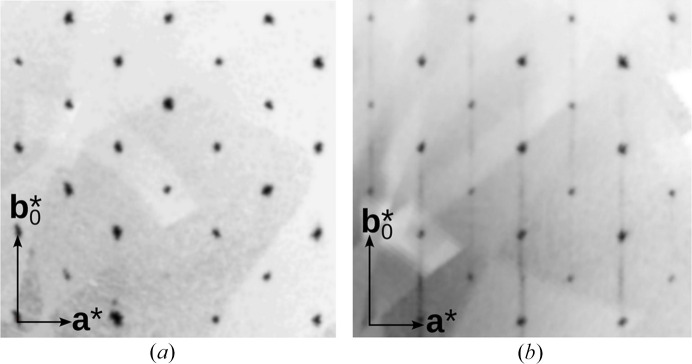
Examples of (*a*) *l* even and (*b*) *l* odd sections through reciprocal space of a K_3_FeTe_2_O_8_(OH)_2_(H_2_O)_2_ crystal reconstructed from a measurement with long exposition times. The indicated reciprocal base is the dual base of (**a**, **b**_0_, **c**).

Faint one-dimensional diffuse scattering perpendicular to (010) (Fig. 2 ▸) clearly indicated a non-negligible stacking fault probability, *i.e.* polytypism, which is consistent with the observed twinning and the disorder of the Te1, Fe1 positions. As noted in the introduction, the OD theory often provides convincing arguments for the polytype character of a structure. The crucial step in an OD interpretation is the identification of the OD layers. This is performed by identifying pseudo-symmetry operations that apply only to a part of the structure. These partial operations (POs) may map layers onto themselves or onto a different layer.

For K_3_FeTe_2_O_8_(OH)_2_(H_2_O)_1+*x*_, the POs correspond to the lost symmetry operations when descending from the *Ibmm* parent structure with the equally disordered Fe1/Te1 position to the *I*2/*m* polytype with two oppositely disordered positions. Since it is known from diffuse scattering along b* that the OD layers extend in the (010) plane, an OD interpretation in terms of two kinds of layers imposes itself as follows.

The K_3_FeTe_2_O_8_(OH)_2_(H_2_O)_1+*x*_ structures are category IV (Grell & Dornberger-Schiff, 1982 ▸) OD structures of two kinds of non-polar (with respect to the stacking directions) layers, named *A* ^1^ and *A* ^2^. The symbol *A* is reserved for non-polar layers and the superscript identifies the kind of the layer (Grell & Dornberger-Schiff, 1982 ▸). The two kinds of layers appear alternately as indicated in Fig. 1 ▸ (right), where the subscript is a sequential number. Note that the exact choice of layer boundary is not relevant as will be shown below and therefore we choose here an interpretation according to crystal chemistry.

**Figure 3:**
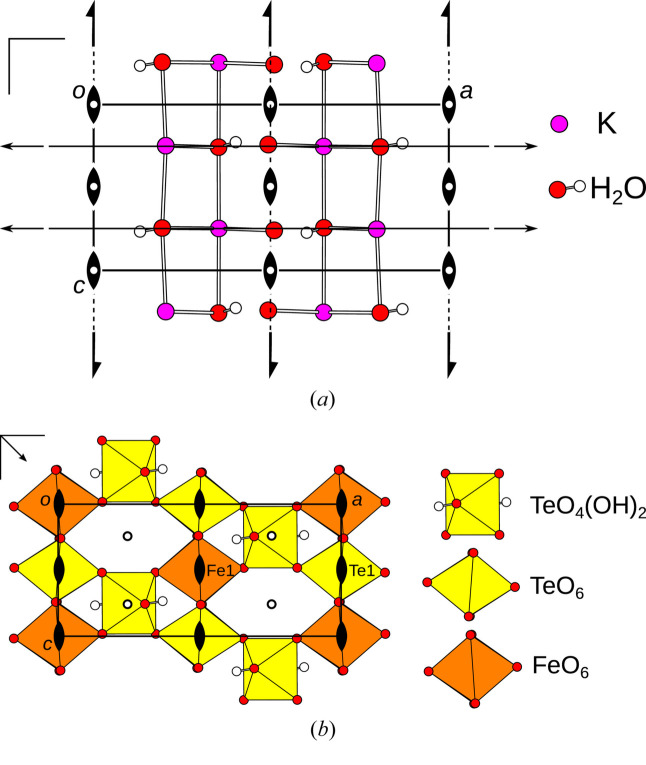
The (*a*) *A* ^1^ and (*b*) *A* ^2^ layers of K_3_FeTe_2_O_8_(OH)_2_(H_2_O)_1+*x*_ projected on the layer plane (010). The idealized symmetry elements according to the OD interpretation are indicated using the usual graphical symbols.

The *A* ^1^ layers comprise a network of K and water molecules [Fig. 3 ▸(*a*)]. The disordered O7 water molecule is not shown, since it is irrelevant for the OD description. The *A* ^2^ layers [Fig. 3 ▸(*b*)] contain the diperiodic FeTe_2_O_8_(OH)_2_ network where intra-layer hydrogen bonds connect the [TeO_4_(OH)_2_] units to the [Te1O_6_] and [Fe1O_6_] octahedra (see O5 atom in Table 2 ▸).

The layers possess *Pc*(*m*)*m* and *P*1(2/*n*)1 symmetry according to the OD interpretation, which, as is typical for OD structures, requires a small degree of idealization. The (pseudo-)symmetry operations are given in Fig. 3 ▸ using the usual graphical symbols. In the OD literature, layer groups are written with capital Bravais symbols to indicate the three-dimensional nature of the layers and with parentheses marking the direction lacking translations. In contrast, the *International Tables for Crystallography* use lowercase Bravais symbols owing to the two-dimensionality of the lattice (Kopsky & Litvin, 2006 ▸). However, the latter symbols imply a stacking direction of [001] and therefore have not found use in the OD literature where alternative stacking directions are often preferable.

The symmetry of a particular OD structure is given by a space groupoid (Ito & Sadanaga, 1976 ▸) of all its POs, which lacks group character, because POs can only be composed if the target layer of the first is the source layer of the second (Ehresmann, 1957 ▸). All OD groupoids of structures built according to the same *symmetry principle* (Fichtner, 1979 ▸) belong to the same OD groupoid family, which can be considered as a generalization of the 230 space group types. The OD groupoid family symbol of K_3_FeTe_2_O_8_(OH)_2_(H_2_O)_1+*x*_ is 
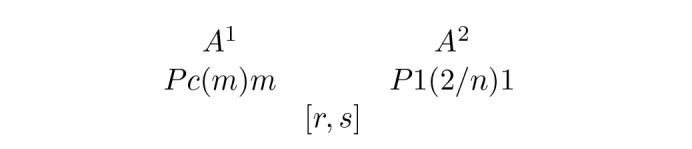
 according to the notation of Grell & Dornberger-Schiff (1982 ▸).

The first line indicates the layer names and the second line the symmetry of the layers. The third line gives the relative position of two adjacent layers in one possible stacking arrangement. [*r*, *s*] means that the origins of the layers are related by *r* **c** + *s* **a** + **b**_0_/2, *r* and *s* being metric parameters in addition to the unit-cell parameters (Fichtner, 1979 ▸). **b**_0_ is the vector perpendicular to the layer planes and the length corresponding to the width of an *A* ^1^ *A* ^2^ layer packet (see Fig. 1 ▸). For K_3_FeTe_2_O_8_(OH)_2_(H_2_O)_1+*x*_, the metric parameters adopt the fixed values 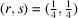 . Indeed, the centers of inversion of the *A* ^2^ layers, which define the origin of its layer group, are located at **c**/4 + **a**/4 + **b**_0_/2 with respect to the centers of inversion of the *A* ^1^ layers (Fig. 3 ▸).

### 3.3. *NFZ* relationship

The stacking possibilities are derived using the *NFZ* (*Z* = *N*/*F*) relationship (Ďurovič, 1997 ▸). In the case of layers of different kinds, the procedure is as follows: the groups of layer operations that do not invert the orientations of the layers with respect to the stacking directions are determined. These groups can be associated with one of the 17 wallpaper group types. One obtains *Pc*(2)*m* for *A* ^1^ and *P*1(2)1 for *A* ^2^, respectively. In a second step, the intersection of the groups is formed, *i.e.* the common operations, called *continuations* in the OD literature, are determined. Here, the values of (*r*,*s*) = 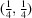 are critical. They cause the 2_[010]_ rotation axes of both layers to overlap perfectly and therefore the group of common operations is *P*1(2)1. Finally, a coset decomposition gives the number and orientations of possible stacking arrangements.

For an *A* ^1^ → *A* ^2^ contact, there are [*Pc*(2)*m*:*P*1(2)1] = 2 cosets and therefore two ways of placing the *A* ^2^ layer. The second possibility is obtained by applying the *c*_[100]_ or equivalently the *m*_[010]_ operation of the *A* ^1^ layer onto the *A* ^2^ layer. This operation exchanges the Te1 and Fe1 positions [Fig. 3 ▸(*b*)]. In contrast, for an *A* ^2^ → *A* ^1^ contact, there is only [*P*1(2)1:*P*1(2)1] = 1 way of placing the *A* ^1^ layer.

### 3.4. MDO polytypes and family structure

According to the OD construction, 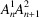 pairs are geometrically equivalent. Likewise, all 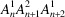 triples are equivalent, since there is only one way of placing the *A* ^1^ layers (see previous section). However, there are two kinds of 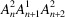 triples, namely those where the *A* ^2^ layers are related by the *m*_[010]_ operation of the central *A* ^1^ layer and those where they are related by the 2_[100]_ operation.

The polytypes containing only one of the two kinds of triples are said to be of a maximum degree of order (MDO) (Dornberger-Schiff & Grell, 1982 ▸). Assuming that the two triples are energetically slightly different, and therefore one is preferred during crystallization, one would assume that ordered polytypes are usually of the MDO kind. Even though a simplistic view, this is indeed very often the case, though exceptions do exist (Nespolo, 2001 ▸; Hybler, 2016 ▸). MDO polytypes are particularly important in an OD interpretation, as all other polytypes can be decomposed into fragments of the MDO polytypes.

**Figure 4:**
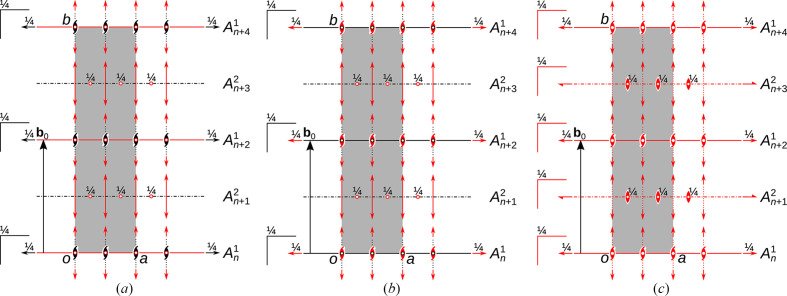
Layer symmetries of the (*a*) MDO_1_, (*b*) MDO_2_ and (*c*) family structure of K_3_FeTe_2_O_8_(OH)_2_(H_2_O)_1+*x*_ according to the OD description. Symmetry elements of the layers are indicated by the usual graphical symbols. Elements that apply to the whole structure are drawn in red. The unit cells are indicated by a grey rectangle.

The layer symmetries of the two MDO polytypes of K_3_FeTe_2_O_8_(OH)_2_(H_2_O)_1+*x*_ are schematized in Figs. 4 ▸(*a*) and 4 ▸(*b*). Operations valid for the whole polytype are indicated in red. The geometric elements of the layers (their symmetry frameworks) are located at the same positions in all polytypes, but the global symmetries differ. The MDO_1_ polytype has *I*2/*m* symmetry, lattice basis vector **b** = 2**b**_0_ in the conventional setting and corresponds to the major polytype of the crystals under investigation. The MDO_2_ polytype has *Pbnn* symmetry with the same unit-cell parameters as MDO_1_, but a primitive lattice. The *P*1(2/*n*)1 symmetry of the *A* ^2^ layers is retained in both polytypes. The idealized *Pc*(*m*)*m* symmetry of the *A* ^1^ layers is reduced to *P*1(2/*m*)1 (MDO_1_) and *P*2(2)2_1_ (MDO_2_), respectively.

The family structure is a fictitious disordered polytype, where all stacking possibilities are realized to the same degree. Its symmetry is schematized in Fig. 4 ▸(*c*). Here the *Pc*(*m*)*m* symmetry of the *A* ^1^ layers is retained and the symmetry of the *A* ^2^ layers increases from *P*1(2/*n*)1 to *Pc*(*n*)*m*. The family structure corresponds to the disordered *Ibmm* structure of the first refinement attempts described above.

Since the point group of the MDO_1_ polytype (2/*m*) is a subgroup of the point group of the family structure (*mmm*) of index 2, stacking faults lead to domains with two different orientions. The domain orientations are related by the point operations of the family structure that are not point operations of the polytype: 2_[100]_, *m*_[100]_, 2_[001]_ and *m*_[001]_. If the domain size is smaller than the coherence length of the radiation, one obtains a disordered structure with higher symmetry. If it is distinctly larger, then the crystal is a twin and the given operations constitute the twin law. If the twin domains are distinctly smaller than the crystal size, the twin volume fractions are approximately equal for statistical reasons. If there are only few stacking faults per crystal, one may obtain twins with unequal volume fractions.

The crystals under investigation were refined at the same time as disordered *and* twins with equal twin volume fractions, which indicates domain sizes in the region of the coherence length (see Table 1 ▸). This is consistent with the observed weak diffuse scattering.

MDO_2_ and the family structure share the same point group, which means that stacking faults in MDO_2_ give domains with the same orientation, but different translation states. Since translations lead to a phase shift of the scattered radiation, these are called *antiphase domains*.

### 3.5. Desymmetrization

Since the actual symmetry of the layers is in general decreased compared to the idealized OD description, one can expect a certain degree of *desymmetrization* (Ďurovič, 1979 ▸), *i.e.* deviation from the idealized symmetry. A full quantification of desymmetrization requires structural data of distinct polytypes (Ďurovič, 1979 ▸), which are not available for the title compounds. One can, however, create an idealized version of the layers with symmetry according to the OD description and quantify the deviation from that idealized structure.

Table 3 ▸ lists the distances of the actual *A* ^1^ layer atoms to the idealized layer. Here, some atoms of the [*M*O_6_] octahedra have been included in the layer, even though crystal-chemically they belong to the *A* ^2^ layers. This is justified by the minute desymmetrization, with the largest deviation of only 0.053 Å. Clearly, the OD description of the *A* ^1^ layers is valid.

The *A* ^2^ layers possess their full symmetry in the MDO polytypes and therefore cannot be idealized. It is nevertheless interesting to quantify the deviation from the *Ibmm* family structure obtained by applying the *m*_[001]_ symmetry, averaging close atoms and moving the atoms onto the *Ibmm* Wyckoff positions (see Table 4 ▸). In this idealized layer, the Te1 and Fe1 atoms share a single position. Apart from that, the deviation from *Pc*(*n*)*m* symmetry is surprisingly minute (max. 0.065 Å). This shows that in principle the whole structure with exception of the Te1 and Fe1 positions adopts the *Ibmm* family structure symmetry, a crucial fact used in the next section to analyze the diffraction pattern. Moreover, it means that the choice of interface between the OD layers is not important as long as the Te1/Fe1 atoms are located in the *A* ^2^ layer.

### 3.6. Diffraction intensities of the MDO polytypes

The diffraction patterns of the title compounds will be described with respect to the basis 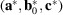 , which is the dual basis of (**a**, **b**_0_, **c**). Since all layers possess a translation lattice spanned by (**a**, **c**), diffraction intensities can only appear on rods 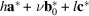 , whereby *h* and *l* are integers, and ν is a real number.

The reflections corresponding to the family structure are called *family reflections* and are always sharp. Those of other polytypes are called *characteristic reflections* and may be more or less diffuse, depending on the degree of order. In many OD structures, the intensities of the family reflections are identical for all polytypes (up to a scaling factor). This is the case if the polytypes can be decomposed into layers that are translationally equivalent, *i.e.* the polytypes differ only in the origin of the layers, but not their orientation. For the title compounds however, the *A* ^2^ layers appear with different orientations and therefore the family reflections may possess different intensities. The diffraction intensities therefore deserve a closer look.

The diffraction patterns of disordered polytypic structures are characterized by a coexistence of discrete and diffuse scattering. Such intensity distributions cannot be expressed using real density functions. Instead, they are properly described by *measures* or *distributions* [see Bricogne (2010 ▸), Baake & Grimm (2013 ▸)]. The classical example of a measure that is not a proper real function is the Dirac measure δ, which associates the weight of 1 to the point *x* = 0. Since the integral of a function that is zero everywhere except at *x* = 0 is 0, δ cannot be a function.

Here, we will disregard such subtleties and use the ‘equivalence’ 

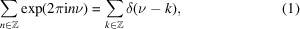

where δ(ν − *k*) is the Dirac measure centered at the point *k*. The equivalence is to be understood in a weak sense, as the function series to the left side does not converge at any point and the right side is not a function.

Since only the positions of the Te1 and Fe1 atoms differ among polytypes, it is useful to consider the contributions of these and the remaining atoms separately. Instead of partitioning the polytypes into two kinds of layers as in the OD description above, it will be described in terms of layers *L*_*n*_ of one kind (Fig. 1 ▸, left side), which are further decomposed into the Te1/Fe1 atoms ( 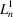 ) and the remaining atoms ( 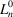 ). The corresponding structure factors are 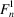 and 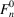 , respectively.

Owing to the *I*-centering of the family structure 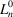 is derived from 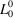 by a translation of *n*(**a**/2 + **c**/2 + **b**_0_). The Te1/Fe1 atoms may additionally be translated by **c**/2 and thus the corresponding translation vector is *n*(**a**/2) + (*n* + *a*_*n*_)**(c**/2) + *n* **b**_0_, where 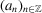 is a bi-infinite sequence with *a*_*n*_ = 0, 1. This sequence fully describes the polytype.

In consequence, the structure factor of the *L*_*n*_ layer is 

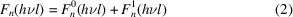

with 

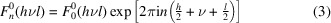

and 

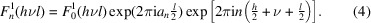

For *l* even, 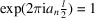 and therefore 

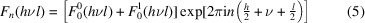

Since this expression is independent of 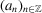 , all polytypes possess the same diffraction pattern on rods *l* even, which consists of sharp reflections corresponding to the family structure. This is in agreement experimental observations [Fig. 2 ▸(*a*)].

Henceforth, only the case *l* odd will be considered and the function arguments *h*ν*l* will be omitted for brevity. Diffraction intensities of polytypes formally calculate as 

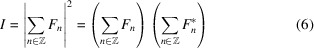




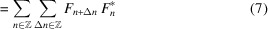




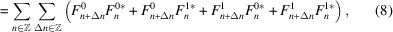

where an asterisk indicates the complex conjugate and it should be stressed again that these function series do not converge at any point. Let us consider the individual terms of equation (8) and introduce the abbreviation φ_Δ*n*_ = 2πΔ*n*[(*h*/2) + ν + (*l*/2)]. The first term is independent of 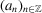 and can be expressed in terms of 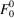 by substituting equation (3) as 

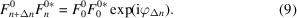

The second and third cross terms depend on 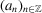 and can be written in terms of 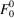 and 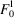 as [see equations (3), (4)]

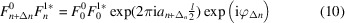




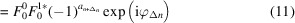

and 

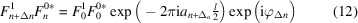




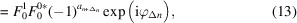

where we used the fact that *l* is odd and *a*_*n*_ = 0, 1. Finally, the forth term depends on the differences *a*_*n*+Δ*n*_ − *a*_*n*_ [see equation (4)]: 

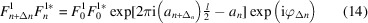




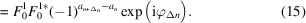

 Combining these terms, the overall intensity *I* can be expressed using two kinds of probabilities. Let *P* be the probability that *a*_*n*_ = 0 for any *n*. Moreover, let *P*_Δ*n*_ be the probability that *a*_*n*+Δ*n*_ = *a*_*n*_. The probabilities are compiled for the MDO polytypes and the family structure in Table 5 ▸. Note that *P* = 0, 1 both correspond to the MDO_1_ polytype, however to the two different twin individuals. Moreover, 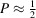 for any structure with a substantial stacking fault probability.

Then, equation (8) becomes 

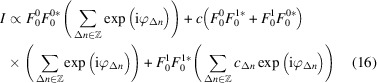




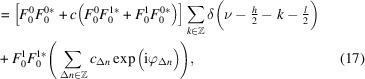

where *c* = 2*P* − 1 and *c*_Δ*n*_ = 2*P*_Δ*n*_ − 1 (*c* being a form of correlation). Thus, there are two distinct contributions to the intensities on rods *l* odd. The first term describes Dirac (Bragg) peaks located at the positions of the family reflections. For MDO_2_, the family structure and substantially disordered stacking arrangements, *c* = 0 (see Table 5 ▸) and thus the reflection intensities are given only by 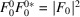 . In contrast, for MDO_1_ the intensity is modified by the cross term 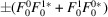 , where the sign depends on the orientation of the twin domain. Note that an expression of the form AB* + B*A is real, but may be negative. Geometrically, it corresponds to the scalar product of *A* and *B* in the complex number plane.

Only the second term of equation (17) with the 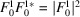 factor may result in diffuse scattering in the case of disordered stacking arrangements, which means that only the Te1/Fe1 atoms contribute to diffuse scattering. The term may however also produce Dirac peaks when the stacking is ordered. In particular, it may add additional intensity to the family reflections. Substituting the *c*_Δ*n*_ value from Table 5 ▸, the 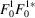 contribution to the diffraction intensities calculates as:

MDO_1_: 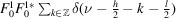 ,

MDO_2_: 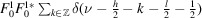 ,

Family structure: 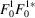 .

For MDO_1_, additional intensity is added to the family reflections, for MDO_2_ additional peaks between the family reflections, as expected given its lack if *I*-centering. The family structure produces an unstructured streak with the form given by 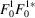 . Even though in general 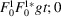 , technically the diffuse scattering of the family structure does not contribute to the intensities of the Dirac peaks. The latter is given as the integral of an infinitesimally area below the peak, which is non-zero for a Dirac peak, but vanishes for a regular distribution (*i.e.* a distribution corresponding to a real function). However, in actual crystals, the Bragg peaks possess a non-zero width owing to experimental artifacts and imperfect crystals and therefore Bragg intensities will be affected by diffuse scattering.

Table 6 ▸ summarizes the diffraction intensities of the MDO polytypes and the family structure on rods *l* odd. The MDO polytypes (two twin domains in case of MDO_1_) and the family structure can be clearly distinguished from Bragg intensities. For MDO_1_, the *L*_*n*_ layers are all translationally equivalent, and therefore the diffraction intensity corresponds to the structure amplitude of a single layer 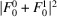 . In the other twin domain, the 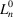 components are unchanged by the twin operation, because *L* ^0^ possesses the point symmetry of the family structure. The exchange of the Fe and Te atoms in the 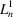 components can also be described by a translation along **c**/2, which corresponds on rods *l* odd to a phase shift of π and thus the structure factor here is 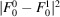 . In MDO_2_ and disordered stacking arrangements, such as the family structure, phases of the 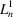 components cancel out systematically or randomly and the structure factor is accordingly 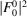 .

**Figure 5:**
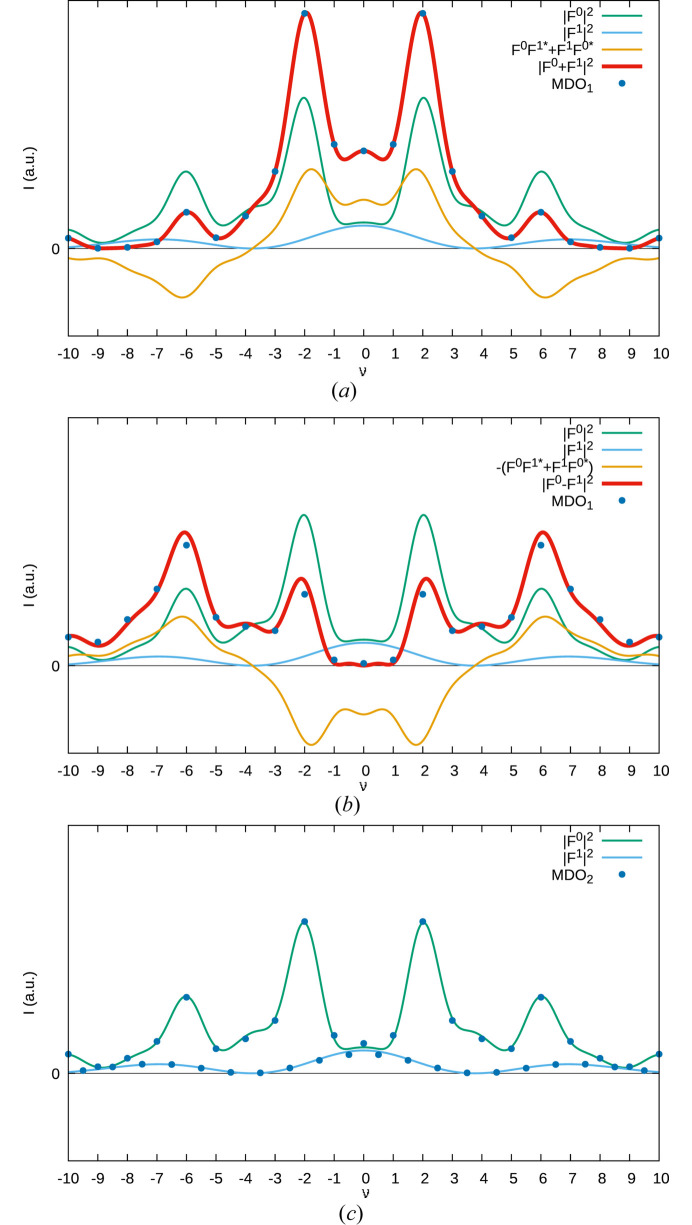
Diffraction intensities of (*a*) MDO_1_, (*b*) its *m*_[100]_ twin domain and (*c*) MDO_2_ on the (1ν3)* rod, represented by blue dots. The relevant factors contributing to the diffraction intensities are given by green ( 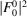 ), blue ( 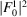 ) and yellow ( 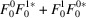 ) curves.

A quantification of the contribution of the different terms is given in Fig. 5 ▸ for the (1ν3)* rod of MDO_1_ (both twin domains) and MDO_2_. The intensities of the polytypes are indicated by blue dots. Note that for MDO_1_ the intensities correspond perfectly to the calculated 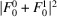 value since MDO_1_ is generated by repeated translation of a single layer. In contrast for the alternative twin domain and MDO_2_ the values differ slightly from 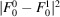 owing to desymmetrization (deviation from *m*_[100]_ symmetry of the actual layers). Nevertheless, the tiny deviation proves the validity of the idealization.

The cross term 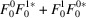 is surprisingly pronounced and changes sign, which makes the MDO polytypes clearly distinguishable based on the intensities of the family reflections. The 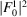 term, which is responsible for the diffuse scattering though is weak. In fact, assuming equal displacement parameters *T* of Te1 and Fe1, 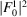 calculates as *T* ^2^|*f*_Te_ − *f*_Fe_|^2^, where *f* stands for the atomic form factors. In other words, the contribution to the diffuse scattering is given by the difference of an Fe and a Te atom (23 electrons).

**Figure 6:**
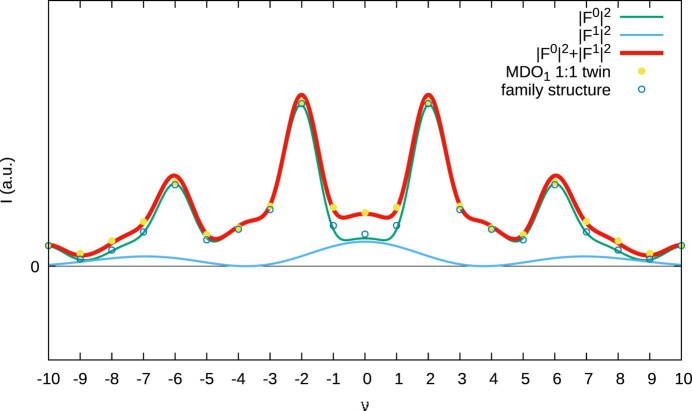
Diffraction intensities of a 1:1 MDO_1_ twin (yellow dots) and the fully disordered family structure (blue circles). The relevant factors contributing to the diffraction intensities are given by green ( 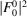 ) and blue ( 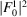 ) curves.

The same small contribution is also the only difference between a twin of MDO_1_ and a fully disordered *Imm*2 structure as shown in Fig. 6 ▸ (also see rows 3 and 5 in Table 6 ▸). Thus, these two models are surprisingly hard to distinguish.

The intensities of the crystal under investigation lie between these two cases, whence the Te1/Fe1 positions had to be modelled as disordered in a ∼ 80 : 20 manner. There must therefore be rather large MDO_1_ domains on the order of magnitude of the coherence length of the employed X-ray radiation. Yet, there must also be rather frequent stacking faults resulting in apparent disorder. This shows a fundamental problem in evaluating such data: the coherence length is not precisely known. In fact, it cannot even be assumed to be a fixed value. In other words, the refined Te1/Fe1 ratio cannot be used to estimate the size of the respective domains as the extent of the twin character (*i.e.* the ratio of coherent and non-coherent diffraction between the domains) is not known.

**Figure 7:**
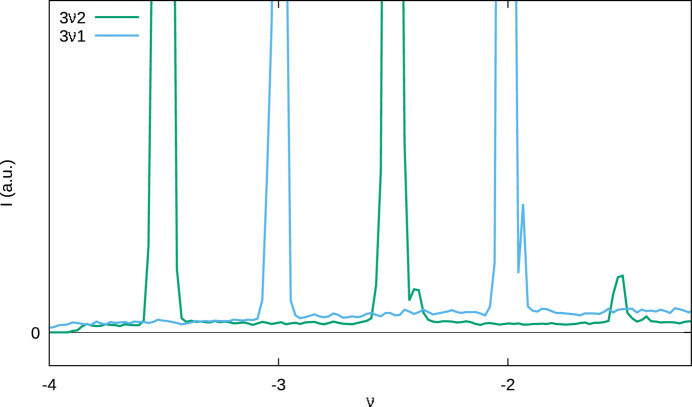
Comparison of the experimental (3ν1)* and (3ν2)* rods of diffuse scattering (compare Fig. 2 ▸). Splitting of the Bragg reflections at ν = −2 and 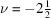 is due to a small secondary domain, also seen in Fig. 2 ▸.

Even though with long exposition times the diffuse scattering is clearly observed [Fig. 2 ▸(*b*)], its intensity is too weak for quantitative analysis (Fig. 7 ▸). This is due to the weak contribution of Fe1/Te1 and because the crystals are at the border between disordered and twinned crystals. The one-dimensional streaks are unstructured, as would be expected for occasional stacking faults.

## 4. Conclusion and outlook

Application of the OD theory has again confirmed its status as ‘the theory of polytypism’ by rationalizing the occurrence of stacking disorder and by classifying the polytype family according to its symmetry principle. However, it remains poorly known in significant parts of the structural science community, even though polytypism is an universal phenomenon. This is certainly due to poor accessibility (*e.g.* lack of software support and standardized notations), but also shortcomings in the theory itself (*e.g.* ambiguities in the choice of layers). Therefore effort should be put into improving the accessibility and the foundation of the theory.

We also showed that single-crystal diffraction in such a case is an unrivaled tool to structurally characterize the average *crystal structure*. When it comes to the *real structure*, however, such as quantification of stacking fault probabilities, complementary methods are required.

## Supplementary Material

Crystal structure: contains datablock(s) 300K, 436K. DOI: 10.1107/S2052520623009162/yv5013sup1.cif

Structure factors: contains datablock(s) 300K. DOI: 10.1107/S2052520623009162/yv5013300Ksup2.hkl

Structure factors: contains datablock(s) 436K. DOI: 10.1107/S2052520623009162/yv5013436Ksup3.hkl

CCDC references: 2301936, 2301937

## Figures and Tables

**Table 1:** Data collection and refinement details

	K_3_FeTe_2_O_8_(OH)_2_(H_2_O)_2_	K_3_FeTe_2_O_8_(OH)_2_(H_2_O)_1.43 (5)_
Crystal data		
Chemical formula	FeH_6_K_3_O_12_Te_2_	FeH_4.86_K_3_O_11.43_Te_2_
*M*_*r*_	626.40	616.17
Crystal system, space group	Monoclinic, *I*2/*m*	Monoclinic, *I*2/*m*
Temperature (K)	300	436
*a*, *b*, *c* (Å)	12.8036 (6), 14.9042 (8), 5.9782 (3)	12.7660 (6), 14.9470 (6), 5.9706 (2)
β (°)	90.002 (2)	90.047 (3)
*V* (Å^3^)	1140.80 (10)	1139.27 (8)
*Z*	4	4
Radiation type	Mo *K*α	Mo *K*α
ρ_calc_ (g cm^−3^)	3.647	3.592
μ (mm^−1^)	7.487	7.491
Crystal size (mm)	0.32 × 0.25 × 0.12	0.18 × 0.08 × 0.01
		
Data collection		
Diffractometer	Bruker KAPPA APEX II	STOE Stadivari
Absorption correction	Multi-scan (*SADABS*)	Multi-scan (*LANA*)
*T*_min_, *T*_max_	0.198, 0.467	0.346, 0.929
No. of measured, independent and observed [*I* > 2σ(*I*)] reflections		
	9677, 2649, 2347	14095, 2711, 2207
*R*_int_	0.0280	0.0431
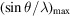 (Å^−1^)	0.83	0.83
		
Refinement		
*R*[*F* ^2^ > 2σ(*F* ^2^)], *wR*(*F* ^2^), *S*	0.0204, 0.0446, 1.012	0.0370, 0.1011, 1.026
No. of parameters	98	98
Δρ_max_, Δρ_min_ (e Å^−3^)	−0.960, 1.331	−1.670, 3.771
Extinction (*SHELXL*)	0.00039 (6)	–
Twin operation	2_[100]_	2_[100]_
Twin volume ratio	53.25 : 46.75 (13)	53.0 : 47.0 (3)
Occupancy Te1:Fe1′	79.2 : 20.8 (2)	79.6 : 20.4 (4)

Computer programs: *X-AREA*, *LANA* (STOE & Cie, 2021 ▸), *SHELXL2014/7* (Sheldrick, 2015*a* ▸), *SHELXT* (Sheldrick, 2015*b* ▸), *OLEX2* (Dolomanov *et al.*, 2009 ▸).

**Table 2:** Hydrogen-bond geometries (Å, °) in K_3_FeTe_2_O_8_(OH)_2_(H_2_O)_2_ at 300 K

	O—H	H⋯O	O⋯O	∠(O—H⋯O)
O5—H1⋯O1	0.87	1.927 (17)	2.723 (2)	151 (3)
O6—H2⋯O4 (2×)	0.87	1.816 (14)	2.653 (3)	161 (4)

**Table 3:** Distances *d* of atoms in the actual *A* ^1^ layer from those in the idealized *A* ^1^ layer, obtained by moving the atoms onto the idealized Wyckoff positions Data derived from the model of K_3_FeTe_2_O_8_(OH)_2_(H_2_O)_2_. The disordered O7 water molecule and the H atoms were ignored.

Atom	Site symmetry	*d* (Å)
K1	.*m*.	0.011
K2	2*mm*	0.017
O4	.*m*.	0.053
O5	.*m*.	0.033
O6	2*mm*	0.042

**Table 4:** Distances *d* of atoms in the actual *A* ^2^ layer to those in the hypothetical *A* ^2^ layers Data derived from the model of K_3_FeTe_2_O_8_(OH)_2_(H_2_O)_2_.

Atom	Site symmetry	*d* (Å)
Fe1/Te1	.2.	0.002
Te2	..2/*m*	0
O1	..*m*	0.065
O2/O3	1	0.012

**Table 5:** Probabilities *P*, *P*_Δ*n*_ and the derived ‘correlations’ *c* = 2*P* − 1 and *c*_Δ*n*_ = 2*P*_Δ*n*_ − 1 for the MDO polytypes, the family structure and disordered structures with a substantial stacking fault probability

	MDO_1_	MDO_2_	Family	Disordered
*P*	0 or 1	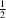	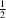	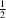
*c*	− 1 or 1	0	0	0
*P*_Δ*n*_	1	0 (Δ*n* odd), 1 (Δ*n* even)	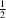 (Δ*n* ≠ 0), 1 (Δ*n* = 0)	Depends
*c*_Δ*n*_	1	(−1)^Δ*n*^	0 (Δ*n* ≠ 0), 1 (Δ*n* = 0)	Depends

**Table 6:** Contributions to Bragg peaks on rods *l* odd

	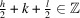 (family reflections)	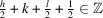
MDO_1_	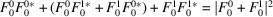	0
MDO_1_ (*m*_[001]_)	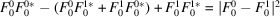	0
1:1 MDO_1_ twin	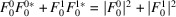	0
MDO_2_	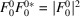	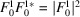
Family structure	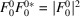	0
